# Protective Role of a New Polysaccharide Extracted from *Lonicera japonica Thunb* in Mice with Ulcerative Colitis Induced by Dextran Sulphate Sodium

**DOI:** 10.1155/2021/8878633

**Published:** 2021-01-02

**Authors:** Xiaonan Zhou, Qingqing Lu, Xiaozhen Kang, Geng Tian, Deguo Ming, Jialiang Yang

**Affiliations:** ^1^School of Basic Medical Sciences, Wannan Medical College, Wuhu, Anhui 241000, China; ^2^NMPA Key Laboratory for Quality Evaluation of Traditional Chinese Medicine, Hangzhou, Zhejiang 310052, China; ^3^Geneis Beijing Co., Ltd., Beijing 100102, China; ^4^Qingdao Geneis Institute of Big Data Mining and Precision Medicine, Qingdao 266000, China; ^5^Shandong Medical Imaging Research Institute, Jinan 250021, China

## Abstract

*Lonicera japonica Thunb* is a traditional Chinese herbal medicine for treating intestinal inflammation. The extraction method of *Lonicera japonica Thunb* polysaccharide (LJP) has been developed previously by our research group. In this study, a Fourier transform infrared spectrometer (FT-IR) was used to perform a qualitative analysis of LJP and a precolumn derivatization high-performance liquid chromatography (HPLC) ((Palo Alto, CA, USA) method was used to explore the monosaccharide composition of LJP. Then, we studied the effect of LJP on the intestinal flora and immune functions of dextran sulfate sodium- (DSS-) induced colitis ulcerative mouse models. The results showed that LJP was consisted of 6 types of monosaccharides and had the characteristic absorption of typical polysaccharides. LJP can increase significantly the weight, organ index, serum cytokines (interleukin, tumor necrosis factor, and interferon-*γ*), secretory immunoglobulin A (SIgA) concentration, and natural killer (NK) cell and cytotoxic lymphocyte (CTL) activities in DSS-treated mice. The results of intestinal flora showed that a high dose (150 mg kg^−1^) of LJP had the best effects on improving the intestinal probiotics (*Bifidobacterium* and *Lactobacilli*) and antagonizing the pathogenic bacteria (*Escherichia coli* and *Enterococcus*). In addition, the measurement results of the spleen lymphocyte apoptosis confirmed from another perspective that LJP had protective effects of immune cells for DSS-treated mice.

## 1. Introduction

Inflammatory bowel disease (IBD) is a refractory chronic bowel disease with two most common forms named Crohn's disease (CD) and ulcerative colitis (UC) [1, 2]. The main clinical symptoms of UC are abdominal pain, diarrhea, and purulent stool. The etiology and pathogenesis of UC are not completely clear, so there is no effective treatment ways [3–5]. Clinically, glucocorticoids and 5-aminosalicylic acid are used for the treatment of UC at present. However, these drugs are difficult to effectively inhibit the deterioration of UC, with severe side effects on the liver function and mental system [6–8]. The clinical symptoms of UC seriously affected the quality of life of the patient.

Alternatively, traditional herbal medicine has continued to be widely used for the prevention and treatment of disease [9, 10]. Previous studies have shown that the activity of herbal medicine can relieve the UC symptoms through exerting their anti-inflammatory potentials. Because of the small side effects, Chinese herbal medicine is being used more and more for the treatment of UC [11–13]. Polysaccharides are one of the basic substances to maintain normal life function. Scientific experiments show that many polysaccharides have biological activities, including immunomodulatory, anti-inflammatory, antibacterial, and antitumor [14–16].

Recent studies reported that some polysaccharides, such as *Polygonatum sibiricum* polysaccharides [17], *Hyriopsis cumingii* polysaccharide [18], and *Ganoderma atrum* polysaccharide [19] have been recommended for potential immunostimulants [20, 21]. *Lonicera japonica Thunb* is a very popular traditional Chinese herbal medicine that can be used to prevent and treat various diseases [22]. *Lonicera japonica Thunb* has been found to have functions of anti-inflammatory, antibacterial, antiallergy, antioxidation, and immunoregulation. *Lonicera japonica Thunb* can be used to treat bacillary dysentery, respiratory infections, high blood pressure, and acute urinary tract infections [23, 24]. *Lonicera japonica Thunb* polysaccharide (LJP) is a major active ingredient in *Lonicera japonica Thunb*. However, few studies reported the effect of LJP on anti-inflammatory and immune function in UC mice. Hence, we aimed to study the effects of LJP on anti-inflammatory and immune functions in UC mice induced by dextran sulphate sodium (DSS) in this study.

*Lonicera japonica Thunb* polysaccharides have been isolated and its characteristics have been described in previous studies by our group. The objective of this study is to further perform a qualitative analysis on LJP, study the monosaccharide composition of LJP, and investigate the anti-inflammatory and immunomodulatory function of the LJP in DSS-induced UC mouse models. The ameliorative effects of LJP were estimated from the weight of the body, spleen and thymus indices, colony-forming units (CFUs) of the cecum, secretory immunoglobulin A (SIgA) of intestinal mucosa, natural killer (NK) cell and CTL activities, tumor necrosis factor (TNF-*α*) and interferon-*γ* (IFN-*γ*) content, serum cytokines (interleukin-2 (IL-2)), and spleen lymphocyte apoptosis in UC mice.

## 2. Materials and Methods

### 2.1. Materials and Chemicals

The laboratory animal procedures were conducted in strict accordance with the National Institutes of Health Guidelines. The animals' care has been approved by the Animal Ethics Committee of the Xuancheng Hospital (SCXK 2019-0007). All the mice were male between 8 and 12 weeks of age. The male mice were placed under standard conditions with plenty of food and water. All efforts were made to minimize suffering. Male Balb/c mice were provided by Shanghai Laboratory Animal Center (Shanghai, China). *Lonicera japonica Thunb* was purchased from Zhongshan Hospital in Maanshan, China, and was deposited in the herbarium of the Wannan Medical College with the voucher number WMC 118162-201905. Fetal bovine serum (FBS) and 3-(4,5-dimethylthiazol-2-yl)-2, 5-diphenyltetrazolium bromide (MTT) were purchased from GIBCO BRL (Grand Island, NY, USA). Standard monosaccharides (D-fucose, D-mannose, D-glucose, D-rhamnose, D-galactose, D-arabinose, and D-xylose), Roswell Park Memorial Institute (RPMI) 1640 medium, dimethyl sulfoxide (DMSO), Annexin V-FITC, CTX, agar medium, and enzyme-linked immunosorbent assay (ELISA) kits for secretory immunoglobulin A (SIgA) IL-2, NF-*α*, and IFN-*γ* were purchased from Novatein Biosciences (Cambridge, MA, USA). YAC-1 and P815 cells were obtained by Cell Bank of Chinese Academy of Science (Shanghai, China).

### 2.2. Preparation of the LJP

*Lonicera japonica Thunb* powder (100 g), 1.5 g of cellulose, and 2 L of distilled water were added in a flask. The mixture was stood for 50 min at 45°C to extract the new polysaccharides from *Lonicera japonica Thunb* powder. After enzyme inactivation (95°C water bath for 15 min), the LJP extracting solution was cooled to room temperature (95°C). Then, the LJP extracting solution was centrifuged at 2655 × g for 10 min. The supernatant of the LJP extracting solution was concentrated to 500 mL and added 2 L 95% (*w*/*w*) ethanol solution. The supernatant was placed at 4°C for 12 hours and then centrifuged at 2655 × g for 10 min. Then, the precipitate was added with 2 mL of chloroform, 10 mL distilled water, and 0.5 mL of n-butanol to remove the protein. Finally, the solution was lyophilized to obtain *Lonicera japonica Thunb* polysaccharides.

### 2.3. FT-IR Measurement Analysis

A Fourier transform infrared spectrometer (IS10, Thermo Nicolet Corporation, Madison, USA) was used to perform a qualitative analysis of LJP. After the LJP sample was lyophilized, 2 mg sample with 100 mg potassium bromide was mixed in it. The mixture was grinded into a uniform powder and then was pressed into slices. The slices were scanned with full-band by an infrared spectrometer (4000-400 cm^−1^). Data were processed using Origin 8.0 software (Origin Lab Corp., Northampton, MA, USA) and OMNIC software (Nicolet, USA).

### 2.4. Monosaccharide Composition Analysis

The precolumn derivatization HPLC method was used to analyze the monosaccharide composition of LJP [25]. First, LJP (10 mg) and 2 mol/L trifluoroacetic acid (TFA, 2.0 mL) were added to the ampoules. The ampoules were sealed, and the LJP was hydrolyzed at 121°C for 1 hour. After hydrolysis, the TFA was evaporated with a vacuum concentrator and the monosaccharide samples were dissolved with distilled water (2.0 mL). Then, the solution was diluted 50 times for HPLC analysis. The monosaccharide component of the LJP was performed on the 2010 edition of the Dionex Ion Chromatograph (Dionex, USA), which contained eluent degassing modules and advanced gradient pumps. The mobile phase consists of acetonitrile and a phosphate buffer (50 mmol/L, pH 6.8), with a volume ratio of 22 : 78. The above conditions were used as the control for standard monosaccharides. The monosaccharide composition of the sample was calculated according to the peak time of the chromatogram. The molar ratio of each monosaccharide was calculated according to the peak area of each chromatographic peak, with that of monosaccharide standards (mannose, arabinose, xylose, galactose, and glucose), respectively.

### 2.5. Experimental Design

The mice (20.4 ± 1.5 g) were randomly divided into the DSS model group, LJP dose groups, and control group. The LJP dose groups included low (LJP-L), medium (LJP-M), and high (LJP-H). The DSS model group and LJP dose group mice were given drinking water with 5% DSS from day 1 to 5 except the control group mice. From day 6 to 15, the mice of LJP dose groups were fed 0.5 mL of LJP aqueous solution. The doses of the LJP-L, LJP-M, and LJP-H groups were 50 mg kg^−1^, 100 mg kg^−1^, and 150 mg kg^−1^ body weight, respectively. Meanwhile, the mice in the control group were given 0.5 mL distilled water daily by gavage. The mice in the DSS model group were given 0.5 mL drinking water with 5% DSS daily by gavage.

### 2.6. Preparation of Mouse Spleen Lymphocyte Suspension

After sacrificing by cervical dislocation under aseptic conditions, the fat and fascia of the mouse spleen were removed using surgical scissors. The spleen was then placed in a 200-mesh sieve in glass culture dish containing 10 mL cold PBS solution. The spleen was gently squeezed with a syringe, and individual cells were passed through the mesh into the solution. The cells were centrifuged at 238 × g for 5 min. After removing the supernatant, red blood cell lysis solution was added to the cells and stood on ice for 5 min. Then, the mixture was centrifuged at 424 × g for 5 min. The precipitate was added with 5 mL RPMI-1640 medium and adjusted to the cell concentration of 2 × 10^5^ mL^−1^.

### 2.7. Measurement of Weight, Spleen Indices, and Thymus Indices

Twenty-four hours after the last gavage, the mice were weighed and sacrificed. The spleen and thymus of mice were gathered and weighted immediately. The calculation formula was as follows: spleen indices (thymus indices) (mg g^−1^) = weight of spleen (thymus) (mg)/weight of mice.

### 2.8. Measurement of Colony-Forming Units (CFUs) of the Cecum

The content of the cecum in mice (1 g) was taken out under sterile conditions and placed in a test tube. The test tube was added with 9 mL sterile saline and shaken for 10 min on a shaker to homogenize the cecum contents. After 10 minutes of standing, the supernatant was further diluted as required using a sterile saline. Diluted samples (1 mL) were inoculated on the culture dish. *Bifidobacterium* and *Lactobacillus* were cultured at 37°C for 48 h (under anaerobic conditions). The trypticase-phytone-yeast (TPY) agar medium and de Man-Rogosa-Sharpe (MRS) agar medium were used as the selective medium. *Escherichia coli* and *Enterococcus* were cultured under aerobic conditions at 37°C for 48 h. The eosin methylene blue (EMB) agar medium and *Enterococcus* selective (ES) agar medium were used as the selective medium. The experiment was repeated three times, and the average values were reported.

### 2.9. Measurement of Secretory Immunoglobulin A (SIgA) of Intestinal Mucosa

After the mice were sacrificed, 5 cm of the small intestine near the ileocecal part was intercepted. Intercepted intestines were tiled on filter paper and dissected longitudinally, and the stool was removed with a razor blade. Then, the mucus on the mucosal surface was scraped with a slide to a 10 mL centrifuge tube. The intestinal mucosal surface was washed repeatedly with 5 mL sterile PBS (0.01 mol L^−1^, pH 7.4), and the washings were collected in 10 mL centrifuge tube. After shaking thoroughly, the tube was centrifuged at 106 × g for 10 min. The supernatant was used for the determination of intestinal mucosa SIgA content with ELISA according to the manufacturer's instructions. The SIgA concentration of the test sample was calculated by substituting the OD value of the test sample into the linear regression equation.

### 2.10. Measurement of NK and CTL Cytotoxicity

The MTT reduction method was used to determine NK and CTL cytotoxicity. According to the principle that the metabolic activity of cells was directly proportional to the number of living cells, MTT assay can measure the decrease of metabolic activity of target cells to reflect the death of target cells caused by effector cells. After entering the cell, oxidized MTT was reduced by mitochondrial dehydrogenase to produce blue granules. Colorimetric quantification after solvent dissolution is directly related to the number of living cells; effector cell killing target cells % can be calculated compared with the target cell control hole. The effector cells (mouse spleen lymphocyte suspension, 2 × 10^5^ mL^−1^) and target cells (YAC-1, 4 × 10^4^ mL^−1^ and P815 cells, 4 × 10^4^ mL^−1^) were seeded into 96-well plates. The ratio of the effector to target cells was 50 : 1. All cells were incubated for 5 h at 37°C in a humidified incubator which contained 5% CO_2_. Then, 20 *μ*L MTT solution (5 mg mL^−1^) was added to continue to culture for 4 h. The supernatants were gathered. In another 96-well plate, 100 *μ*L supernatants were added to each well. Then, DMSO (50 *μ*L) was added to each well and shook for 20 min. The optical density (OD) was detected at 490 nm by a microplate reader. The killing activity is calculated by killing activity = [Target cell − (Effect − target cell OD value − Effects OD value)/Target cell OD value] × 100%.

### 2.11. Measurement of Serum Cytokine IL-2, TNF-*α*, and IFN-*γ* Content

The eyeballs of mice were taken out, and the blood was collected from the eye socket. The collected blood was put into the EP tube. After standing for 2 hours at room temperature, the blood was centrifuged at 1301 × g for 15 min. The upper serum was used to detect the serum cytokine IL-2, TNF-*α*, and IFN-*γ* content by the ELISA kits in the light of the instruction of the manufacturer.

### 2.12. Measurement of Spleen Lymphocyte Apoptosis

In the flow test tube, 300 *μ*L mouse spleen lymphocyte suspension (2 × 10^5^ mL^−1^) was added and centrifuged at 424 × g for 6 min. The precipitate was added with 500 *μ*L binding buffer. Then, the tube was added with 5 *μ*L Annexin V-FITC and 5 *μ*L propidium iodide (PI). The tube was blended and placed at a dark place for 10 min. The apoptotic cells were detected by a flow cytometry (FACS Aria II, NJ, USA).

### 2.13. Statistical Analysis

The analysis of variance (ANOVA) was performed by SPSS 20.0 software (IBM, Chicago, IL, USA). The significant difference between mean values was deterred by one-way analysis of variance. A 95% confidence level (*P* < 0.05) was considered to be statistically significant.

## 3. Results

### 3.1. FT-IR Analysis of LJP

**Figure 1:**
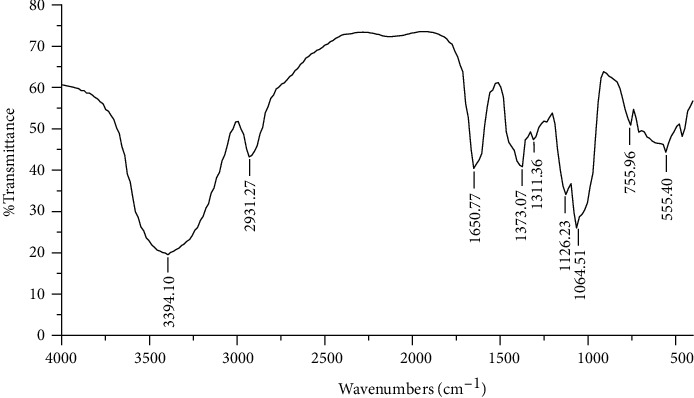
FT-IR spectrum of LJP in the range of 4000–400 cm^−1^.

The FT-IR spectrum of LJP is shown in Figure 1 in the range of 4000–400 cm^−1^. LJP had two typical absorption peaks of sugar compounds. The first characteristic absorption peak was between 3600 and 3200 cm^−1^. This characteristic absorption peak was caused by the vibration of the O-H bonds in the sugar compound. The second characteristic absorption peak was around 2900 cm^−1^. This characteristic absorption peak was caused by the vibration of the C-H bonds in the sugar compound [17]. In addition, there was a characteristic absorption peak at 1650 cm^−1^ which was caused by the crystalline water of a carbohydrate compound. There were three stretching peaks at 1311, 1126, and 1064 cm^−1^. These were attributed to the presence of C-O glycoside bonds [26, 27]. The above results showed that LJP had typical polysaccharide absorption characteristics.

### 3.2. Monosaccharide Composition of LJP

The monosaccharide composition of LJP is shown in Table 1, which mainly consisted of glucose (Glu), arabinose (Ara), rhamnose (Rha), mannose (Man), xylose (Xyl), and galactose (Gal). The composition of monosaccharides is the basis of the chemical structure of LJP, and there is a certain correlation with the biological activity of polysaccharides. Exploring and understanding the monosaccharide composition of polysaccharides helps to better utilize and develop LJP.

### 3.3. Effect of LJP on the Weight, Spleen Indices, Thymus Indices, and SIgA of UC Mice

**Figure 2:**
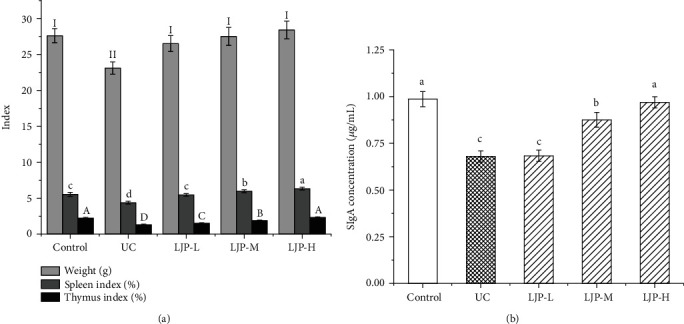
Effect of LJP on the weight, spleen index, thymus index, and SIgA of UC mice induced by DSS. Results are the mean ± standard deviation. Statistical significance was determined by one-way analysis (ANOVA). Roman letters (I, II, and III) versus control groups for weigh. Lowercase letters (a, b, c, and d) versus the control groups for the spleen index and SIgA. Uppercase (A, B, C, D, and E) versus the control groups for thymus index.

As can be seen from Figure 2(a), there was no significant difference in the initial body weight between different groups (*P* > 0.05). There was no significant difference in body weight between the LJP group and control group (*P* > 0.05). The weight of the UC group was significantly lower than that of the control group (*P* < 0.05). The spleen and thymus indices of the DSS-treated group were reduced significantly compared with the control samples (*P* < 0.05). The spleen and thymus indices of mice in LJP-H doses also rose significantly than that of LJP-L doses (*P* < 0.05). These results indicated that LJP can increase the weight of immune organs in UC mice.

With the different concentrations of the standard sample as the abscissa and OD (450 nm) as the ordinate, a standard curve was drawn and the linear regression equation was *y* = 0.1256*x* − 0.0328 with a correlation coefficient of *R*^2^ = 0.9937. The results are shown in Figure 2(b). The SIgA concentration of UC mice was significantly lower than that of the control group (*P* < 0.05). There was no significant difference in the SIgA concentration between the LJP-L group and UC mice. The SIgA concentrations in the LJP-M and LJP-H dose groups were significantly higher than those in the UC mice (*P* < 0.05).

### 3.4. Effect of LJP on the CFUs of Cecum

As can be seen in Table 2, the CFUs of *Bifidobacterium* and *Lactobacillus* in UC mice were significantly lower than those in the control group (*P* < 0.05). The CFUs of *Escherichia coli* and *Enterococcus* in UC mice were significantly higher than those in the control group (*P* < 0.05). These results indicated that the mouse intestinal flora has been disrupted and the UC mouse model induced by DSS was successfully established. The LJP-L group had no effect on the CFUs of *Bifidobacterium* in UC mice, but the CFUs of *Bifidobacterium* of the LJP-M and LJP-H dose groups were significantly higher than those in the UC group (*P* < 0.05). The CFUs of *Lactobacillus* in the LJP-L, LJP-M, and LJP-H dose groups were significantly higher than those in the UC group (*P* < 0.05). The LJP-L group did not decrease the CFUs of *Escherichia coli* in UC mice, but the CFUs of *Escherichia coli* in the LJP-M and LJP-H groups were significantly lower than those in the UC group (*P* < 0.05). The CFUs of *Escherichia coli* in the LJP-H dose group had been reduced to the level of the control group. The LJP-L group had no effect on the CFUs of *Enterococcus* of UC mice, but the CFUs of *Enterococcus* in the LJP-M group had reduced at the level of the control group.

### 3.5. Effect of LJP on NK and CTL Cytotoxicity and IL-2, TNF-*α*, and IFN-*γ* concentrations of UC Mice

**Figure 3:**
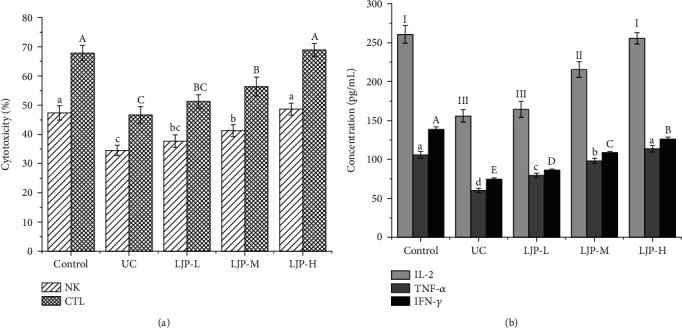
Effect of LJP on the NK and CTL cytotoxicity and the serum IL-2, TNF-*α*, and IFN-*γ* concentrations of UC mice induced by DSS. Results are mean ± standard deviation. Statistical significance was determined by one-way analysis (ANOVA). Lowercase letters (a, b, and c) versus the control groups for NK and TNF-*α*. Uppercase letters (A, B, and C) versus the control groups for CTL and IFN-*γ*. Roman letters (I, II, and III) versus the control groups for IL-2.

Figure 3(a) showed the effects of LJP on NK and CTL cytotoxicity in mice fed LJP. Under the influence of DSS, the NK and CTL cytotoxicity was significantly reduced in the UC group (*P* < 0.05). With the improvement of the LJP dose, the NK and CTL cytotoxicity of mice increased gradually. In the LJP-H dose group, the NK and CTL cytotoxicity reached a level that was not significantly different as the control group.

Figure 3(b) showed that, under the influence of DSS, the serum concentrations of IL-2, TNF-*α*, and IFN-*γ* were significantly decreased in the UC group (*P* < 0.05). After feeding LJP, the serum concentrations of IL-2, TNF-*α*, and IFN-*γ* in UC mice increased gradually with the increase of LJP doses. At the low-dose group (LJP-L), the serum concentrations of IL-2, TNF-*α*, and IFN-*γ* in UC mice were not significantly different from those in the control group. At the high-dose group (LJP-M and LJP-H), the serum concentrations of IL-2, TNF-*α*, and IFN-*γ* in UC mice were significantly higher than those in the UC group (*P* < 0.05).

### 3.6. Effect of LJP on Spleen Lymphocyte Apoptosis

**Figure 4:**
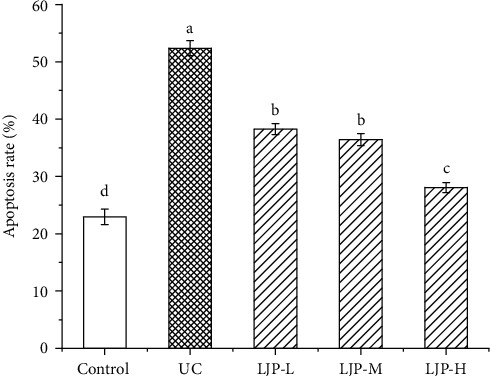
Effect of LJP on the spleen lymphocytes apoptosis in UC mice induced by DSS. Results are mean ± standard deviation. Statistical significance was determined by one-way analysis (ANOVA). Lowercase letters (a, b, and c) versus the control groups.

Figure 4 showed the experimental results of the spleen lymphocyte apoptosis rate. The apoptosis rate in the UC group increased by 128% compared with the control group. LJP can remarkably inhibit the raise of the spleen lymphocyte apoptosis rate in UC mice induced by DSS. With the increase of the LJP concentration, the apoptosis rate of spleen cells gradually returned to normal. However, compared with the control group, there was still a gap of 22% in the apoptosis rate.

## 4. Discussion

Over 2.5 million residents in Europe and 1 million in the USA are estimated to have IBD. China has a population exceeding 1.4 billion people. With expanding urbanization and westernization, the number of IBD cases in China could at some point overtake the number of cases in the Western world [28]. UC is one of a major type of inflammatory bowel diseases (IBD). Aminosalicylic acid and hormones are the major existing therapies, and many undesirable side effects were found during the treatment of UC. Hence, it is urgently needed to search the better therapeutic agents with no side effects [29–31].

Recently, many studies have found that polysaccharides extracted from plants and fungus can activate immune cells, inhibit tumor growth, and improve the body's immune function [32–34]. In a previous study, a new polysaccharide from *Lonicera japonica Thunb* had been isolated and characterized by us. In this study, mice were given drinking water with 5% DSS to establish a UC mouse model. Lee et al. [35] found that BuOH extracts of *Lonicera japonica* reduced the crypt injury and may be useful as a potential inhibitor for preventing inflammatory bowel disease in humans. The experimental results showed that DSS remarkably reduced the body weight; organ indices; the levels of SIgA, NK, and CTL cytotoxicity; and the serum IL-2, TNF-*α*, and IFN-*γ* concentrations of mice. DSS also caused disorders in the intestinal flora of mice. Moreover, the apoptosis rate of spleen lymphocytes increased 128% in UC mice. The above data demonstrated that the successful establishment of a UC mouse model with DSS was consistent with some previous studies [36, 37].

The weight of the body, spleen, and thymus are the body's innate immune function indicators [38]. The spleen is a peripheral immune organ. After maturing, the immune cells will colonize in the spleen and respond to the antigen. The thymus is a central immune organ. The main function of the thymus is to produce T lymphocytes. Therefore, the changes of the spleen and thymus indices reflect the strength of body's innate immune function [39]. In our experiments, LJP significantly increased the weight of the body, spleen, and thymus in DSS-treated mice. These results indicated that intestinal disorders caused by DSS affected the gastrointestinal digestion and absorption function of mice in the UC model group, which caused weight loss. After being given LJP by gavage, the gastrointestinal digestion and absorption functions of mice were gradually restored and the weight of mice also increased.

A multitude of bacteria live in the intestine of healthy animals. These bacteria play a very crucial role in participating in the metabolism of nutrients and in the composition of the gut immune system. Changes in intestinal flora are one of the causes of UC. Many studies have shown that the structure and quantity of bacteria have changed significantly in UC patients [40, 41]. In our experiments, the numbers of *Bifidobacteria* and *Lactobacilli* in the intestine of UC mice were significantly decreased compared with the control group, and the numbers of *Escherichia coli* and *Enterococci* were significantly increased. After the treatment with LJP, the numbers of *Bifidobacteria* and *Lactobacilli* in the intestine of UC mice were significantly increased, and the numbers of *Escherichia coli* and *Enterococci* were significantly decreased. These results indicated that LJP can regulate the intestinal disorder caused by DSS to a certain extent and restored the gut flora. Among them, a high dose (150 mg kg^−1^) of LJP had the best effects on improving the intestinal probiotics (*Bifidobacterium* and *Lactobacilli*) and antagonizing the pathogenic bacteria (*Escherichia coli* and *Enterococcus*).

SIgA is an immunoglobulin secreted by plasma cells of the intestinal mucosa and a major effector of the intestinal mucosal immunity. SIgA is the first line of defense in the intestinal mucosa that neutralizes pathogens in the intestinal mucosa and plays an important role in local anti-infection of the body [42, 43]. The experimental study found that DSS can inhibit the secretion of intestinal SIgA. After being given LJP by gavage, the intestinal SIgA content of mice was significantly higher than that of UC mice. These results showed that LJP can promote the secretion of SIgA in the mouse intestinal mucosa. Intestinal flora and SIgA interact in the intestine. *Bifidobacterium* proliferation can promote the secretion of SIgA. And vice versa, SIgA maintains normal intestinal flora balance by immunization [44].

NK and CTL are two major populations of cytotoxic lymphocytes in the immune system of the body. NK and CTL are not only directly connected to immune regulation but also involved in the autoimmune diseases [45]. CTL specializes in the secretion of various cytokines involved in immune function [46]. Under the absence of the major histocompatibility complex (MHC), NK can also directly recognize and kill the infected cells [47]. In this study, under the influence of DSS, the NK and CTL cytotoxicity was significantly reduced in the UC group. After feeding with LJP, the NK and CTL cytotoxicity of mice increased gradually. These results demonstrated that LJP can enhance the intestinal immune function in UC mice.

Cytokines are a class of small molecule proteins with a wide range of biological activities secreted by immune cells and certain nonimmune cells. Cytokines can stimulate hematopoietic function, participate in the development of immune cell differentiation, mediate inflammatory reaction, and regulate immune response [48]. IL-2 can promote the proliferation and differentiation of cells and induce the production of interferon [49]. TNF-*α* has a direct killing effect on tumor cells, which plays a very crucial role in the host defense mechanism. In addition, TNF-*α* can also enhance the expression of a series of inflammatory mediators and immune mediators [50]. The major biological activities of IFN-*γ* in serums are to induce the expression of MHC molecules, inhibit viral replication, and activate macrophages [51]. From the experimental results of the LJP groups, we can find that LJP had a good regulatory effect to the secretion of serum cytokines in UC mice. After feeding LJP, the serum concentrations of IL-2, TNF-*α*, and IFN-*γ* in UC mice increased gradually with the increase of LJP doses. These results show that LJP can improve the symptoms of low immunity and regulate the body's immune imbalance.

There are two main types of cell death. One is cell necrosis, and the other is apoptosis. Apoptosis is the autonomous and orderly death of cells controlled by genes under certain physiological or pathological conditions. As one of the basic characteristics of life, apoptosis and cell proliferation together maintain the internal environment stability of body [52]. Disorders of apoptosis will trigger tumor and immune system diseases [53, 54]. In this study, apoptosis of splenic lymphocytes was detected by flow cytometry. The results indicated that the balance of lymphocyte apoptosis in DSS-induced UC mice was disrupted, and the apoptosis rate was increased by 128%. After being given LJP by gavage, the apoptosis rate of spleen lymphocytes was gradually restored. This result indicated that LJP can attenuate the apoptosis of splenic lymphocytes in UC mice.

According to the results of this study and the above discussion, it was concluded that LJP had typical polysaccharide absorption characteristics and consisted of 6 types of monosaccharides. LJP can improve the symptoms of low immunity in UC mice induced by DSS. These biochemical and cytological experimental results indicated that LJP can restore the immune disorder and raise the anti-inflammatory activities of immune organs in UC mice. The present results may be helpful for further clinical application of LJP.

## Figures and Tables

**Table 1:** Monosaccharide compositions of LJP.

	Mol%					
	Glu	Gal	Man	Rha	Xyl	Ara
LJP	61.37	8.29	2.73	5.39	2.58	19.64

**Table 2:** Effect of LJP on the CFUs of the cecum in UC mice induced by DSS (log CFU g^−1^).

Groups	*Bifidobacterium*	*Lactobacillus*	*Escherichia coli*	*Enterococcus*
Control	10.35 ± 0.25^a^	11.55 ± 0.61^a^	7.86 ± 0.36^c^	6.37 ± 0.29^c^
UC	8.31 ± 0.34^d^	8.25 ± 0.49^d^	9.68 ± 0.63^a^	7.95 ± 0.61^a^
LJP-L	8.95 ± 0.36^cd^	9.68 ± 0.36^c^	9.07 ± 0.75^ab^	7.37 ± 0.53^ab^
LJP-M	9.47 ± 0.28^bc^	10.25 ± 0.48^bc^	8.61 ± 0.28^b^	6.88 ± 0.33^bc^
LJP-H	9.86 ± 0.33^ab^	10.96 ± 0.56^ab^	8.13 ± 0.34^bc^	6.54 ± 0.71^c^

Results are mean ± standard deviation. Statistical significance was determined by one-way analysis (ANOVA). Lowercase letters (a, b, c, and d) versus the control groups. Different lowercase letters in the same column within the same sample express the significant differences (*P* < 0.05).

## Data Availability

The data generated during the present study are available from the corresponding author on reasonable request.
